# Sodium Alginate Hydrogel Sponges Embedded with M2 Macrophages: An Adoptive Cell Therapy Strategy for Accelerated Diabetic Wound Healing

**DOI:** 10.3390/gels11070502

**Published:** 2025-06-27

**Authors:** Qingchang Tian, Wenqi Li, Lijiaqi Zhang, Kefen Gan, Yiting Zhang, Shuling Wang

**Affiliations:** 1 School of Pharmacy, Hangzhou Normal University, Hangzhou 311121, China; 2 Key Laboratory of Elemene Class Anti-Cancer Chinese Medicines, Hangzhou Normal University, Hangzhou 311121, China; 3 Engineering Laboratory of Development and Application of Traditional Chinese Medicines, Collaborative Innovation Center of Traditional Chinese Medicines of Zhejiang Province, Hangzhou Normal University, Hangzhou 311121, China

**Keywords:** hydrogel sponges, wound healing, M2 macrophage, cell therapy

## Abstract

Hydrogels possess advantages for providing a moist wound environment and enabling drug or cell delivery. Wound healing is a complex, multistage process where macrophages play a pivotal role; they influence inflammation resolution, anti-inflammatory cytokine production, angiogenesis, and extracellular matrix remodeling. Combining hydrogel materials with adoptive M2 macrophages offers a promising adoptive cell therapy approach to accelerate healing. In the present study, sodium alginate, a natural polymer, was harnessed to create hydrogel sponges embedded with M2 macrophages for application to chronic wounds. Hydrogel sponges were capable of preserving the characteristics of M2 macrophages, secreting functional cellular factors, and maintaining viability. Hydrogel sponges loaded with M2 type macrophages could promote chronic wound healing in the back of type 2 diabetic mice. In vitro and in vivo experiments demonstrated that M2 macrophages successfully grew and proliferated within the hydrogel sponges, exhibiting anti-inflammatory effects, which is expected to offer a cell therapy approach to diabetic wound treatment.

## 1. Introduction

Diabetes, a complex metabolic disease, frequently impairs and delays wound healing, often leading to the development of debilitating and painful skin ulcers [1]. These ulcers pose a significant economic burden on patients [2]. The failure of wound re-epithelialization remains a major challenge in diabetic wound care. Current treatment strategies include wound debridement, skin grafting, antibiotic therapy, and the use of tissue engineering sponges [3]. Additional therapeutic interventions involve negative pressure therapy, hyperbaric oxygen therapy, and the application of growth factors [4]. Wound management for diabetic patients represents an urgent social issue, necessitating the development of novel and effective therapies.

Macrophages exhibit significant phenotypic plasticity and diversity, making them the primary effector cells of innate immunity and key mediators of tissue regeneration [5]. In addition, it has been established that alternatively activated macrophages (M2), which are considered anti-inflammatory and promote regeneration, play important roles in wound healing [6]. During the initial stages of normal wound healing, M1 macrophages actively remove surrounding debris through exocytosis, secrete pro-inflammatory mediators, and exhibit antimicrobial functions. Subsequently, during the repair phase, macrophages transition into an anti-inflammatory M2 phenotype, promoting tissue regeneration by influencing key aspects of wound healing such as angiogenesis, extracellular matrix remodeling, production of anti-inflammatory cytokines, and the resolution of inflammation [7,8]. Wounds lacking macrophages result in delayed re-epithelialization processes, impaired angiogenesis, reduced collagen deposition, and decreased cell proliferation [9]. In chronic diabetic wounds, dysregulation between the pro-inflammatory and anti-inflammatory phenotypes of macrophages persists, with a constant state of M1 activation, long-term presence of an unrestrained inflammatory pattern associated with chronic wounds, reduced production of key anti-inflammatory cytokines and growth factors hindering the development of damaged tissues into the proliferation and remodeling phases of wound healing [10,11]. In addition, macrophages have been identified as an important therapeutic target for accelerating chronic wound healing in diabetes [12], and modulating the balance between M1 and M2 polarization states of macrophages represents an effective means to regulate chronic wound healing [13]. Building upon the above findings, a novel glycopeptide hydrogel with the composition of glycoproteins and nanofiber structure was designed to mimic the extracellular matrix of skin cells, inducing mannose receptor activation to polarize macrophages into the M2 phenotype. The polarization of macrophages accelerates chronic wound healing without the requirement of other therapeutic interventions [14]. Similarly, researchers have recently developed glycopeptide hybrid hydrogels capable of promoting macrophage M2 polarization inspired by the chemical composition, fiber structure, and biological function of natural extracellular matrix, remodeling damaged tissues by coordinating abundant M2 macrophages to reduce inflammation and promote angiogenesis [15].

Adoptive cell transfer therapy employing macrophages offers a safer therapeutic tool in cancer therapy [16]. M2 macrophages to the wound site represents a feasible strategy for rapidly accelerating the transition from the inflammatory stage to the proliferative repair stage. External adoptive M2 macrophages may also secrete pro-inflammatory mediators and reshape the microenvironment. The direct application of adoptive cells by loading these cells onto hydrogels can provide a suitable microenvironment for wound healing. This approach minimizes potential damage associated with traditional injection methods. Additionally, the three-dimensional environment provided by hydrogels facilitates cell–cell and cell–matrix interactions, which helps prevent abnormal cellular behaviors such as changes in morphology, polarity, and division methods [17]. Alginate (Alg), in tissue engineering is promising, as they can deliver cells to desired sites and promote new tissue formation [18]. Its biocompatibility, liquid absorption capacity and low cytotoxicity make alginate hydrogel a versatile material in biomedical and engineering fields for drug and cell delivery [19,20,21].

**Figure 1:**
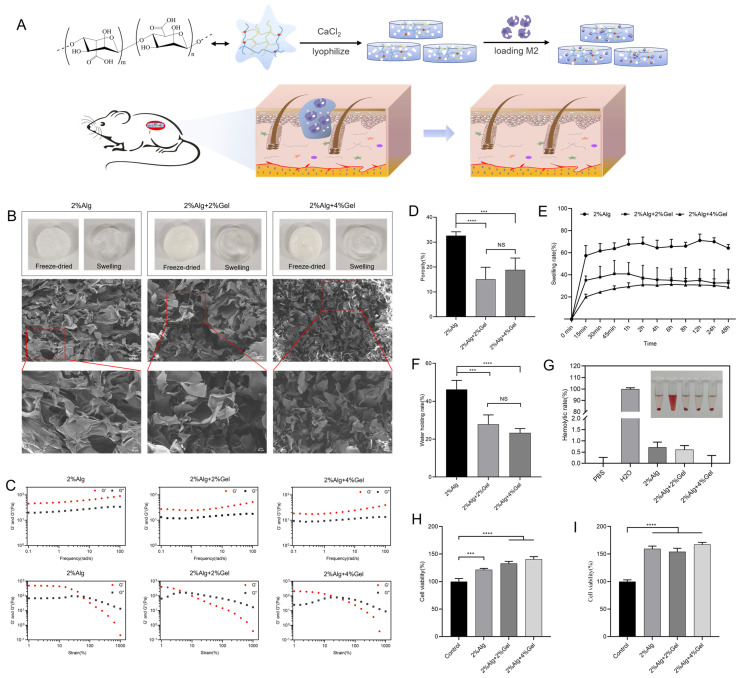
Characterization of hydrogel sponges. (**A**) Illustration of hydrogel sponges loaded with M2 macrophages for application at skin defects to exert a positive effect on promoting wound healing. (**B**) SEM images of hydrogel sponge. The scale bars are 100 μM (up) and 30 μM (down). (**C**) Rheological properties of hydrogels. (**D**) Porosity of hydrogels, (*n* = 3, mean ± SD). *** *p* < 0.001, **** *p* < 0.0001, and NS for no significant difference. (**E**) Swelling curves of hydrogels. (**F**) Water holding capacity of hydrogels, (*n* = 3, mean ± SD). *** *p* < 0.001, **** *p* < 0.0001, and NS for no significant difference. (**G**) Blood compatibility of hydrogels. (**H**,**I**) Cell compatibility of hydrogels at 24 h and 48 h, (*n* = 3, mean ± SD). *** *p* < 0.001, **** *p* < 0.0001, and NS for no significant difference.

Based on the design outlined above, we hypothesized that M2 macrophage-loaded hydrogel sponges would address the issue of wound healing. Herein, we presented an adoptive cell transfer strategy employing M2 Macrophages-loaded hydrogel sponges for cell therapy of wound healing. Importantly, we sought to design a hydrogel sponge loaded with M2 macrophages that could be applied directly to skin defects to promote wound healing (Figure 1A). While contemporary research on chronic wound repair predominantly involves functional hydrogel dressings, stem cell application, or cytokine delivery, the use of adoptive M2 macrophage-loaded hydrogel sponges directly on chronic wounds represents an innovative approach to accelerate healing.

## 2. Results and Discussion

### 2.1. Characterization of Hydrogel Sponges

Three kinds of hydrogel sponges were prepared and lyophilized and fractured for scanning electron microscopy (SEM) analysis. SEM analysis confirmed that 2%Alg, 2%Alg + 2%Gel, and 2%Alg + 4%Gel formed typical microporous structures with internal cavities and pore sizes of approximately 110 μM, 100 μM, and 50 μM, respectively (Figure 1B). Rheological studies demonstrated that the hydrogel sponges exhibited gel-like behavior within the frequency range of 0.1–100 rad/s. Increased strain could disrupt the hydrogel network structure, causing a transition from a gel state to a sol state (Figure 1C). Porosity is a critical parameter influencing metabolism, nutrient transport, and gas exchange within hydrogel sponges. Interconnecting pores provide a suitable microenvironment for cell adhesion, proliferation, and differentiation. 2%Alg demonstrated the highest porosity at 32%. However, the addition of gelatin decreased the porosity to 15% and 18% (Figure 1D). The 2%Alg sponge also exhibited the strongest swelling behavior, reaching equilibrium after 2 h (Figure 1E). Similarly, 2%Alg showed the highest water retention, significantly exceeding the capacity of 2%Alg + 2%Gel and 2%Alg + 4%Gel hydrogel sponges (Figure 1F). Sterility examinations revealed no bacterial growth in hydrogel-sponge extracts over seven days, confirming sterility (Figure S1). All three hydrogel sponges (2%Alg, 2%Alg + 2%Gel, and 2%Alg + 4%Gel) demonstrated good blood compatibility without causing hemolysis (Figure 1G). Cell compatibility tests indicated no significant toxicity toward fibroblasts after 24 and 48 h of exposure (Figure 1H,I). Live/dead staining further corroborated the non-toxic nature of the hydrogels at 24 and 48 h (Figures S2 and S3).

### 2.2. In Vitro Evaluation of the Hydrogel Sponges

**Figure 2:**
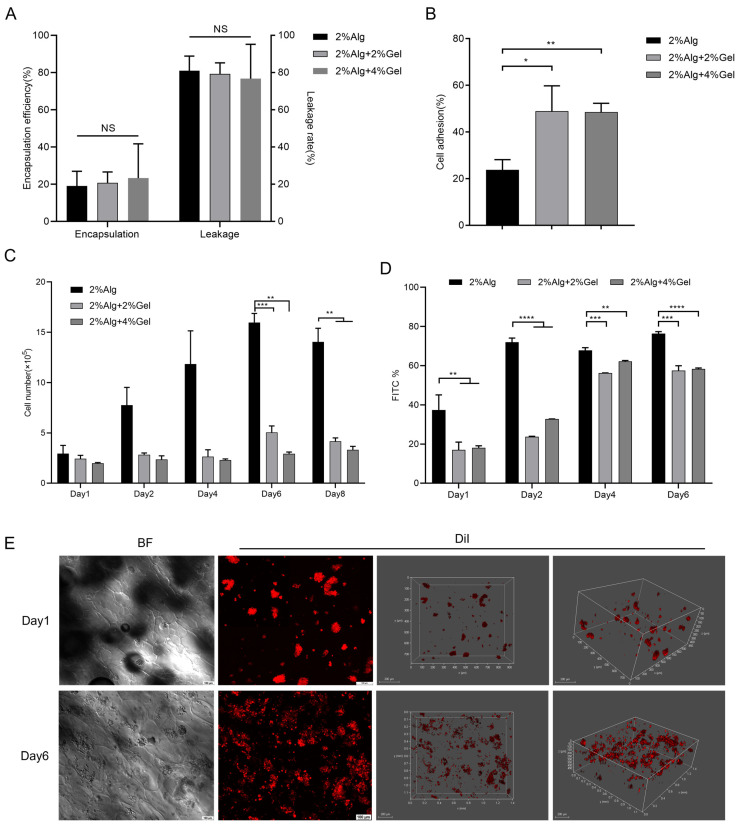
In vitro evaluation of the hydrogel sponges. (**A**) The encapsulation rates of M2 macrophages on the three hydrogel sponges (*n* = 3, mean ± SD), NS for no significant difference. (**B**) The adhesion rates of M2 macrophages on hydrogel sponges. (*n* = 3, mean ± SD), * *p* < 0.05, ** *p* < 0.01, and NS for no significant difference. (**C**) The proliferation of M2 macrophages on the hydrogel sponges. (*n* = 3, mean ± SD), ** *p* < 0.01, *** *p* < 0.001. (**D**) The viability of M2 macrophages in hydrogel sponges. (*n* = 3, mean ± SD), ** *p* < 0.01, *** *p* < 0.001, **** *p* < 0.0001. (**E**) The distribution of M2 macrophages within 2%Alg hydrogel. The scale bars are 100 μM (**right**) and 200 μM (**left**).

We first confirmed successful polarization of M0 macrophages to M2 phenotype after 48 h of treatment with IL-4 at 20 ng/mL (Figure S4). The average encapsulation rates of M2 macrophages in the 2%Alg, 2%Alg + 2%Gel, and 2%Alg + 4%Gel sponges were 19%, 21%, and 22%, respectively, showing no statistically significant differences among groups (Figure 2A). After washing with PBS, the average adhesion rates of M2 macrophages increased to 23.8%, 48.9%, and 48.5%, respectively (Figure 2B). Notably, the introduction of gelatin significantly improved cell adhesion, likely due to the presence of Arg-Gly-Asp (RGD) sequences in gelatin that provide cell attachment sites. Initially, there was no significant difference in the abundance of M2 macrophages across the groups (Figure 2C). However, over the culture period, M2 macrophages in the 2%Alg group exhibited significantly higher proliferation, reaching approximately three times their initial number by day 6. This was significantly higher compared to the 2%Alg + 2%Gel and 2%Alg + 4%Gel groups. Flow cytometry analysis also revealed the strongest green fluorescence (indicating live cells) in the 2%Alg group throughout the 6-day culture period, significantly higher than the other groups, substantiating that hydrogel sponges could serve as a three-dimensional growth carrier for cells (Figure 2D and Figure S5).

We employed the encapsulation rate, adhesion rate, proliferation rate, and survival rate of M2 macrophages as criteria to screen the optimal approach for embedding cells within lyophilized hydrogel sponges. While the encapsulation rate displayed no significant difference across the three hydrogel sponges, the introduction of gelatin significantly improved M2 macrophage adhesion but reduced sponge porosity, which could potentially hinder the exchange of nutrients and waste products, consequently affecting cell growth and proliferation. Both cell counting and flow cytometry analyses revealed that the 2%Alg hydrogel group exhibited the best performance in terms of cell proliferation and survival. Considering the above factors, we selected the 2%Alg hydrogel sponge as the optimal material for embedding M2 macrophages and employed it in subsequent experiments.

We investigated the distribution of M2 macrophages within the 2%Alg hydrogel using 3D stereoscopic imaging. The results revealed even cell distribution along the sponge edge, extending approximately 400 μM within the hydrogel. Furthermore, the cell number increased significantly from days 1 to 6 (Figure 2E).

**Figure 3:**
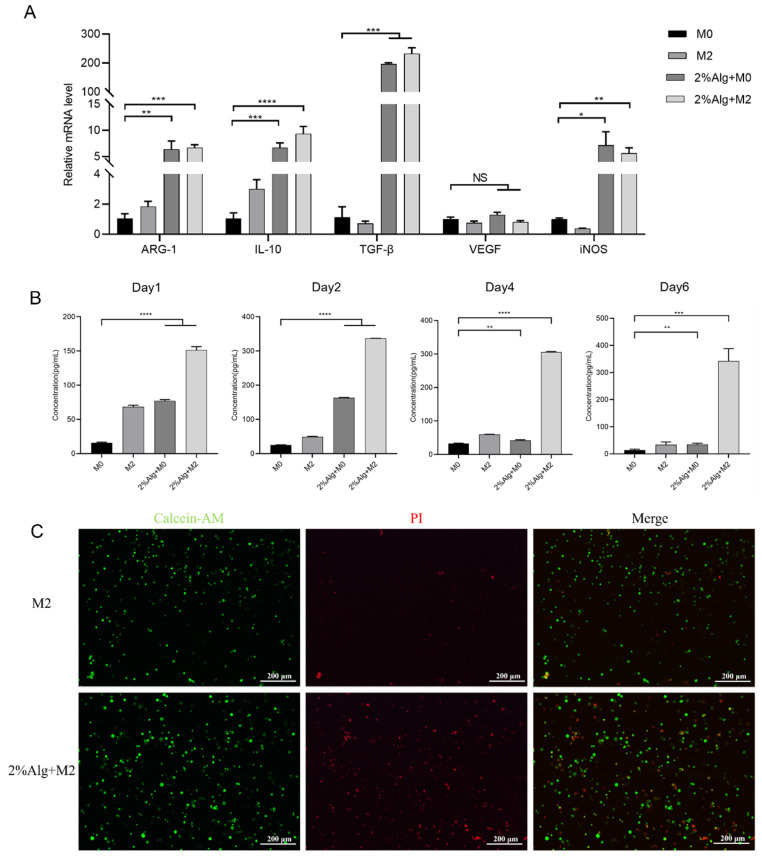
The characteristics of M2 macrophages in hydrogel sponges. (**A**) The gene expression levels of *iNOS*, *Arg-1*, *IL-10*, *TGF-β*, and *VEGF*, (*n* = 3, mean ± SD), * *p* < 0.05, ** *p* < 0.01, *** *p* < 0.001, **** *p* < 0.0001 and NS for no significant difference. (**B**) The secretion of VEGF by M2 macrophages cultured for 6 days in the hydrogel, (*n* = 3, mean ± SD), ** *p* < 0.01, *** *p* < 0.001, **** *p* < 0.0001. (**C**) The viability of cryopreserved M2 macrophages encapsulated in the hydrogel after one week. The scale bars are 200 μM.

RT-qPCR detection of gene expression levels of *iNOS*, *Arg-1*, *IL-10*, *TGF-β* and *VEGF* was performed to demonstrate the wound healing ability of hydrogel sponges encapsulating M2 macrophages. Interestingly, M0 and M2 macrophages cultured on the sodium alginate hydrogel exhibited high expression of both the M2 marker Arg-1 and the M1 marker iNOS, suggesting a possible partial conversion of M2 to M1 phenotype due to environmental influences within the sponge. Albeit M1 marker expression was observed, compared to uncultured M0 macrophages, both M0 and M2 macrophages cultured on the hydrogel sponge significantly upregulated the expression of IL-10, TGF-β, and VEGF within 4 days, with IL-10 and TGF-β remaining highly expressed on day 6 (Figure 3A and Figure S6). Enzyme-linked immunosorbent assay (ELISA) confirmed the enhanced secretion of VEGF by M2 macrophages cultured for 6 days in the hydrogel compared to control groups (Figure 3B). Finally, we tested the viability of cryopreserved M2 macrophages encapsulated in the hydrogel after one week. Live/dead cell staining revealed successful revival of most entrapped macrophages (Figure 3C).

The findings suggest that Hydrogel sponges are capable of preserving the characteristics of M2 macrophages, secreting functional cellular factors, and maintaining viability when stored in liquid nitrogen for extended periods. These attributes lay a solid foundation for their potential utilization in subsequent applications.

### 2.3. In Vivo Wound Healing Evaluation of the Diabetic Wound Model

To evaluate their ability to heal chronic wounds, 2%Alg hydrogel sponges embedded with M2 macrophages were tested in a full-thickness skin defect model in type 2 diabetic mice.

**Figure 4:**
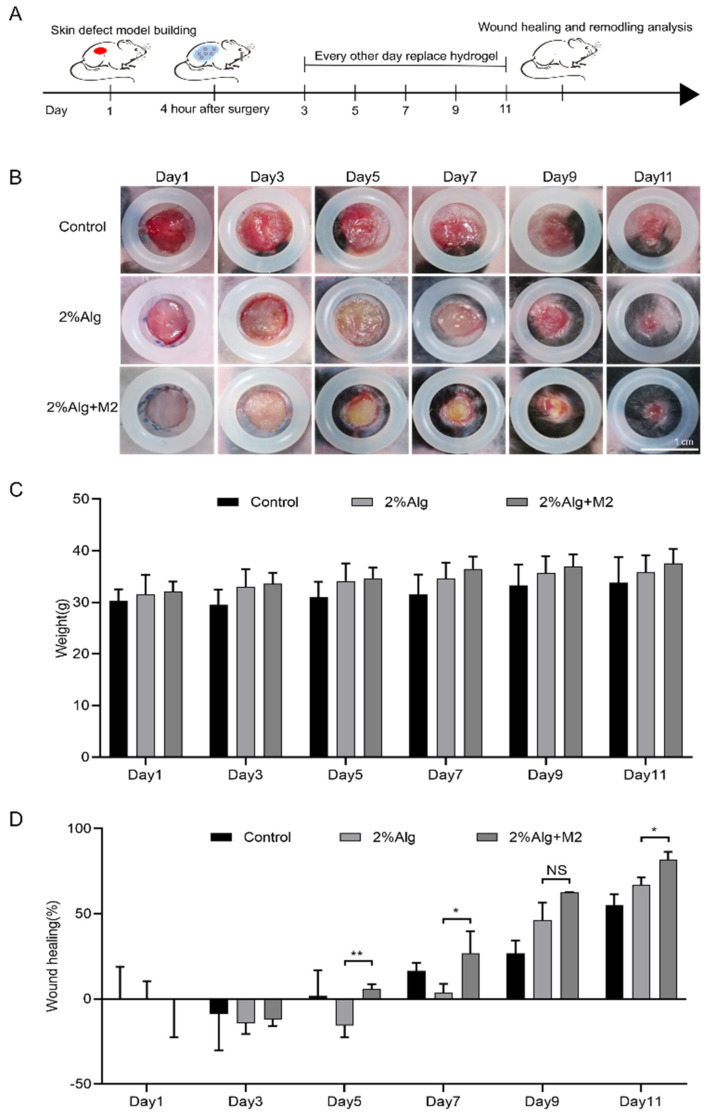
In vivo wound healing evaluation. (**A**) Treatment schedule for evaluation of Hydrogel sponges. (**B**) Optical photography of the wound. The scale bar is 1 cm. (**C**) Body weights of mice in different groups (*n* = 3, mean ± SD). (**D**) wound healing rate of mice, (*n* = 3, mean ± SD). * *p* < 0.05, ** *p* < 0.01, and NS for no significant difference.

Photographs of the wounds of mice were obtained at various time points following hydrogel sponge treatment (Figure 4A). After 5 days, the 2%Alg + M2 group exhibited significantly smaller wounds compared to the Control and 2%Alg groups. By day 11, wounds in the 2%Alg + M2 group were essentially healed, demonstrating significantly accelerated closure compared to other groups (Figure 4B). Analysis of weight changes during treatment (Figure 4C) revealed significant weight gain in the 2%Alg + M2 group (* *p* < 0.05), whereas mice in the Control and 2%Alg groups showed no significant weight change. This suggests that M2-type macrophage-encapsulated sodium alginate hydrogel treatment promotes healing without adverse effects on overall health. ImageJ (Version 1.53e) analysis revealed an 82 ± 4.4% wound closure rate in the 2%Alg + M2 group after 11 days, exceeding both the Control (55.2 ± 6.2%) and 2%Alg (67 ± 4.3%) groups. The significantly higher healing rate in the 2%Alg + M2 group demonstrates the efficacy of the M2-type macrophage-encapsulated sodium alginate hydrogel sponge in promoting full-thickness skin excision wound healing in diabetic mice (Figure 4D).

**Figure 5:**
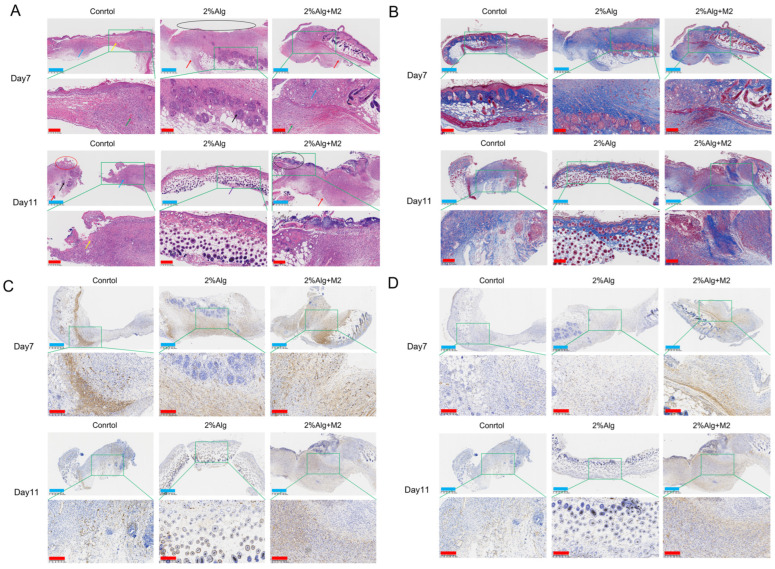
The wound healing was characterized by histological section analysis. (**A**) HE staining. (**B**) Masson staining. (**C**) Immunohistochemical staining with CD31 antibody. (**D**) Immunohistochemical staining with α-SMA antibody. The scale bars are 625 μm (**blue**) 200 μm (**red**).

Histological analysis further characterized wound healing. After 11 days of treatment, HE and Masson staining revealed significant surface collagen deposition, granulation tissue formation, and epidermal healing in the 2%Alg + M2 group, outperforming the 2%Alg group (Figure 5A,B). Sirius Red (SR) staining demonstrated a predominance of red type I collagen in the 2%Alg + M2 group with a small amount of yellow-green type III collagen arranged in an organized, compact network (Figures S7 and S8). Neovascularization is a crucial indicator of wound healing. CD31 (platelet-endothelial cell adhesion molecule) is a widely used marker for endothelial cells lining blood vessels. We assessed the formation of new blood vessels during chronic skin wound healing by immunohistochemical staining for CD31. As shown in Figure 5C, significant angiogenesis was observed in the 2%Alg + M2 group on days 7 and 11, while minimal angiogenesis was found in the Control and 2%Alg groups (Table S1). H-Score quantification further confirmed higher CD31 expression in the 2%Alg + M2 group compared to the 2%Alg group, indicating increased endothelial cells within newly formed blood vessels. Alpha-SMA, a myofibroblast marker, plays an essential role in wound contraction. Positive alpha-SMA expression was detected in all groups (Control, 2%Alg, and 2%Alg + M2) as shown in Figure 5D (Table S2). H-Score scoring revealed higher alpha-SMA expression in the 2%Alg + M2 group compared to the 2%Alg group, suggesting increased formation of mature blood vessels in the wound area.

To further support the findings of pathological analysis, we examined the expression levels of IL-10, IL-6, TNF-α, TGF-β, VEGF, Col3a1, and Col1a1 by RT-qPCR. On days 7 and 11 of the healing process, the 2%Alg + M2 group exhibited significantly higher expression of IL-10 and TGF-β, while IL-6 and TNF-α expression were significantly lower. VEGF expression peaked on day 7 and returned to normal levels by day 11. Additionally, Col1a1 and Col3a1 expression was significantly higher on day 7 (Figures S9 and S10).

## 3. Conclusions

In this study, we prepared lyophilized hydrogel sponges for the treatment of chronic skin wounds. The hydrogel sponges served as three-dimensional carriers for M2 macrophages. The 2%Alg hydrogel sponge exhibited an interconnected channel structure, high porosity, excellent swelling, and biocompatibility. M2 macrophages successfully grew and proliferated within the 2%Alg hydrogel sponge, maintaining their original functions and phenotypes. When applied to wounds on the backs of type II diabetic mice, the M2 macrophage-embedded hydrogel sponge significantly reduced wound area and promoted healing. However, after several days of culture within the sponge, M2 macrophages expressed both the M2 marker Arg-1 and the M1 marker iNOS. This suggests the possibility of partial phenotypic transformation of M2 macrophages in the hydrogel environment, an aspect warranting further investigation. This highlights a limitation of the study, as current detection methods only provide an overall picture of cell phenotype and function, and further research is needed to address this solicitude.

In addition, we successfully prepared M2 macrophage-loaded hydrogel sponges. We demonstrated that M2 macrophages can thrive and maintain their anti-inflammatory properties within these hydrogel sponges. Furthermore, application of these hydrogel sponges promoted wound healing in diabetic mice, suggesting a promising new approach for diabetic wound treatment.

## 4. Materials and Methods

### 4.1. Material

Sodium alginate (Mw = 198.11 kDa) was acquired from Aladdin (Shanghai, China). Calcein-AM/PI, Double Stain Kit, 1,1′-Dioctadecyl-3,3,3′,3′-tetramethylindocarbocyanine perchlorate (Dil) were all purchased from YEASEN (Shanghai, China). Primers of target genes mRNA were custom synthesized by Tsingke Biotechnology Co., Ltd. (Beijing, China). The details of the primer sequences used are listed in Table S3. All oligonucleotides were dissolved in sterile water and stored at –20 °C. AxyPrep^TM^ Multisource Total RNA was purchased from AXYGEN (Santa Clara, CA, USA). Lipopolysaccharide (LPS), 4′,6-diamidino-2-phenylindole (DAPI), type B gelatin, brain heart infusion medium dry powder and agar powder were obtained from Solarbio (Beijing, China). Interleukin-4 (IL-4) was purchased from PERPROTECH (Cranbury, NJ, USA). Cell Counting Kit-8 (CCK-8) was obtained from Dalian Meilun Biotechnology Corporation (Dalian, China). TB Green Premix Ex Taq II was procured from Takara (Dalian, China). The antibodies (Table S4) used for immunofluorescence were obtained from Abcam (Cambridge, UK) and Proteintech (Rosemont, IL, USA). Mouse VEGF ELISA Kit (Table S4) was purchased from Beyotime (Shanghai, China).

The mouse macrophage cell line RAW264.7 was obtained from the Chinese Academy of Science Cell Bank for Type Culture Collection (Shanghai, China). High-glucose DMEM supplemented, fetal bovine serum and penicillin/streptomycin were obtained from ExCell Bio (Shanghai, China).

### 4.2. Preparation and Characterization of Hydrogel Sponges

#### 4.2.1. Preparation of Hydrogel Sponges

Preparation of the following three solutions: 2% sodium alginate solution (2%Alg), 2%Alg with 2% type B gelatin (2%Alg + 2%Gel), and 2%Alg with 4% type B gelatin (2%Alg + 4%Gel). To form the hydrogels, each solution was mixed with a 5 mM CaCl_2_ solution in a 1:1 ratio, stirred thoroughly at 1000 rpm for 5 min, and then dispensed into 24-well plates (1 mL per well). After freezing overnight at −80 °C, the samples were freeze-dried. Subsequently, the freeze-dried hydrogels were crosslinked again with a 10 mM CaCl_2_ solution, followed by another freeze–thaw cycle (−80 °C) and freeze-drying. Finally, the freeze-dried samples were sealed for storage until future use.

#### 4.2.2. Internal Morphology Observations

Three lyophilized hydrogel sponges were fractured for scanning electron microscopy analysis. The hydrogel sponges were frozen in liquid nitrogen for 5 min to achieve a brittle state, facilitating fracture and cross-sectional exposure. Subsequently, the fracture surfaces were gold-sprayed for enhanced conductivity, and observed under SEM at an accelerating voltage of 3 kV.

#### 4.2.3. Rheological Properties

Freeze-dried hydrogel sponges were fully immersed in ultrapure water to reach equilibrium swelling. Subsequently, the hydrogel sponges’ mechanical properties were evaluated using a rheometer. A frequency sweep test was conducted at a fixed strain level of 1% within the frequency range of 1–100 rad/s. Additionally, a strain sweep test was performed at a constant frequency of 1 Hz, applying a strain range of 1% to 1000%.

#### 4.2.4. Porosity

The porosity of the three hydrogel sponges was determined using the liquid displacement method. The volume (V) and weight (W_1_) of the freeze-dried hydrogel sponges were measured before immersion in anhydrous ethanol. The hydrogel sponges were then submerged in anhydrous ethanol until saturated. After removing the hydrogel sponges, excess ethanol on the surface was gently wiped off with filter paper, and the final weight (W_2_) was recorded. In the equation below, ρ_0_ is the density of anhydrous ethanol.

Porosity (%) = (W_2_ − W_1_)/(ρ_0_V)

#### 4.2.5. Swelling Property

The three hydrogel sponges were freeze-dried and their initial weights were recorded as W_0_. Subsequently, the freeze-dried samples were immersed in PBS (pH 7.4) at 37 °C. At predetermined time points, the samples were removed from the PBS. Excess surface water was carefully removed using filter paper, and the final weights were recorded as W_t_. The swelling ratio was then calculated as the average weight change (W_t_ − W_0_) normalized by the initial weight (W_0_) and expressed as a function of time.

Swelling property (%) = (W_t_ − W_0_)/W_0_

#### 4.2.6. Water Holding Capacity

After the cross-linking of three hydrogel sponges was completed, the surface liquid was absorbed by filter paper after washing with ultrapure water, and the wet weight of the hydrogel sponge was weighed as W_1_. The hydrogel sponge was freeze-dried in a freeze dryer for 24 h, and the dry weight of the hydrogel sponge was measured as W_2_, and the water holding capacity of the hydrogel sponge was calculated.

Water retention (%) = (W_1_ − W_2_)/W_2_

#### 4.2.7. Sterility Examination

Following Standard ISO 10993 [22], we prepared extraction solutions considering the solvent absorption properties of the freeze-dried hydrogel sponges. The volume of extraction medium absorbed per 1.0 cm × 1.0 cm of freeze-dried sponge was determined beforehand. During material extraction, this absorption volume was added to the extraction solution mixture for every 1.0 cm × 1.0 cm of material being extracted. Therefore, after ultraviolet disinfection, three types of hydrogel sponges (2%Alg, 2%Alg + 2%Gel, and 2%Alg + 4%Gel) were incubated with PBS (37 °C, 5% CO_2_, 100% humidity) for 24 h in a culture box. To account for solvent absorption, 1 mL of extraction medium was added per extractable area of each sponge. The resulting extraction solutions were stored at 4 °C for further use.

A total of 9.25 g of brain heart infusion medium dry powder and 0.925 g of agar powder were combined, followed by the addition of 250 mL of ultrapure water to dissolve the mixture. The solution was then autoclaved at 121 °C for 15 min. After cooling the solution to 50–60 °C, it was poured into plates. Once the plates cooled and solidified, they were coated and incubated at 37 °C for several days to assess bacterial growth. Three experimental groups were established: one containing the hydrogel extract, a negative control group with PBS, a positive control group with tap water, and a blank group without any reagent.

#### 4.2.8. Blood Compatibility

The preparation method of hydrogel sponge extract was described in Section 4.2.7. Fresh blood from BALB/C mice was washed with saline and centrifuged at 3000 rpm for 5 min until the supernatant was transparent, and the obtained precipitate was mouse live RBCs. The RBCs were resuspended in saline to a final concentration of 5% (*v*/*v*). Saline was set as negative control; ultrapure water was set as positive control; three kinds of hydrogel extracts were set as experimental groups. After being placed in an incubator at 37 °C for 3 h, they were centrifuged at 2000 rpm for 10 min and photographed. The supernatant was added to a 96-well plate, and the absorbance of each well at 546 nm was read using an ELISA reader, and the hemolysis rate was calculated according to the following formula:

Hemolysis rate (%) = (OD experimental group − OD negative group)/(OD positive group − OD negative group)

#### 4.2.9. In Vitro Cytocompatibility

##### Preparation of Fibroblasts

Skin samples from C57BL/6 mice were placed in the culture dish, and a small amount of medium was added to ensure firm adherence. The culture medium consisted of high-glucose DMEM supplemented with 10% fetal bovine serum (volume fraction). The culture conditions were maintained at 37 °C in a 5% CO_2_ atmosphere. After 24 h, an adequate volume of culture medium was added. The medium was changed only once during the first week. After one week, when primary cells had fully covered the culture bottle, the primary tissue block was removed, trypsinized for digestion, and passaged for subculture.

##### CCK-8 Assay for Detection of In Vitro Cytocompatibility of Hydrogel Sponges

The preparation method for the culture medium leachate of the three hydrogel sponges is described in Section 4.2.8.

Murine fibroblasts were seeded into a 96-well plate at a density of 1 × 10^4^ cells per well. Then, 100 μL of the extract was added to each well as the treatment group. Following incubation for 24 and 48 h, the supernatant in the wells was removed, and 10% CCK-8 solution was added to each well. The cultures were incubated in the dark for 1 h, and the optical density (OD) at 450 nm was measured using an ELISA reader. The relative cell proliferation rate was calculated using the following formula:

Relative cell proliferation (%) = At/Ac

where At is the absorbance of cell culture wells with added hydrogel extract, and Ac is the absorbance of culture wells with added DMEM.

##### Live/Dead Assay to Detect In Vitro Cytocompatibility of Hydrogel Sponges

As described in Section 4.2.8, culture medium leachate was prepared from the three hydrogel sponges.

Mouse fibroblasts were seeded in small dishes and treated with the extract as the experimental group. Following incubation for 24 and 48 h, the cells were washed twice with 1× Assay Buffer for 2 min per wash. Subsequently, AM/PI cell viability staining solution was added, and the cells were incubated in the dark at room temperature for 30 min. After washing with PBS buffer two to three times, the cells were visualized under a fluorescence microscope to assess live cells (yellow-green fluorescence) and dead cells (red fluorescence).

### 4.3. In Vitro Study of M2 Macrophage-Hydrogel Sponges

#### 4.3.1. Polarization of Macrophage M2 Type

M0 macrophages were serum starved for 1 h and cultured with RPMI 1640 supplemented with 10% FBS, while IL-4 solution (final concentration of 20 ng/mL) was added to induce M2 macrophages after 48 h.

#### 4.3.2. Cell Encapsulation Rate

Three hydrogel sponges were placed in non-tissue culture-treated 24-well plates, and M2 macrophage suspension (400 μL/well containing 5 × 10^5^ cells) was added and incubated for 1 h. After that, each hydrogel sample was digested with 50 mM EDTA solution, the cells were collected and counted using a cell counting plate, and the cell encapsulation rate and leakage rate were calculated.

#### 4.3.3. Cell Adhesion Rate

Three types of hydrogel sponges were placed in non-tissue culture-treated 24-well plates. Each well received 400 μL of M2 macrophage suspension containing 5 × 10^5^ cells. After incubation for 1 h in an incubator, 1 mL of culture medium was added to each well, and the cultures were maintained under normal conditions. Following 16 h of incubation, the culture medium was discarded. The hydrogel sponges were then removed and rinsed once with PBS solution. Subsequently, the hydrogel sponges were digested with a 50 mM EDTA solution to release the cells. The collected cells were then counted using a cell counting plate, and the cell adhesion rate was calculated.

#### 4.3.4. Cell Proliferation Rate

The hydrogel sponges (5 × 10^5^ cells) embedded with M2 macrophages were taken out after culturing for 1, 2, 4, 6, and 8 days. The hydrogel samples were digested with 50 mM EDTA solution and the cells were collected to count using a cell counting plate, and the cell proliferation rate was calculated.

#### 4.3.5. Cell Growth

After 1, 2, 4, and 6 days of incubation with the hydrogel sponge containing M2 macrophages, the hydrogel sponge was digested with a 50 mM EDTA solution to extract cells. After washing with PBS 1~2 times, AM/PI staining solution was added and incubated at room temperature in the dark for 30 min. After washing with PBS for 1~2 times, it was resuspended, and cell fluorescence was observed by flow cytometry.

#### 4.3.6. Cell Distribution

Macrophages were stained with DiI cell membrane fluorescent probe (final concentration 5 μM) and incubated at 37 °C for 30 min. DiI-stained M2 macrophages were seeded onto hydrogel sponges (5 × 10^5^ cells), cultured in an incubator for 1 h, supplemented with 1 mL of culture medium, and then normally cultured. Cell fluorescence was observed on days one and six using laser confocal microscopy.

#### 4.3.7. Expression of Cell Marker Genes

After culturing the 2%Alg hydrogel sponge with M2 macrophages for several days, the hydrogel sponge was digested with a 50 mM EDTA solution to extract cells, and RNA was extracted by kit to detect the gene expression of Arg-1, VEGF, IL-10, TGF-β, and iNOS in cells by RT-qPCR.

#### 4.3.8. Expression of Cytokine VEGF

After 1, 2, 4, and 6 days of culture in the 2%Alg hydrogel sponges containing M2 macrophages, cell supernatants were collected and the concentration of target proteins in the samples was detected according to the instructions of the Mouse VEGF ELISA Kit (Table S4).

#### 4.3.9. Cryopreservation and Recovery of M2 Macrophage-Hydrogel Sponges

Harvested, normally cultured M2 macrophages were resuspended in a cryoprotectant solution. Drops of the cell suspension were then added to the 2%Alg hydrogel. The hydrogel with encapsulated cells was transferred to cryovials, sealed and placed in an isopropanol cell cryopreservation box. After overnight storage in an ultra-low temperature refrigerator at −80 °C, the cryovials were transferred to liquid nitrogen for long-term storage.

One week later, the hydrogel sponges with embedded M2 macrophages were retrieved from liquid nitrogen and placed in culture medium. The medium was changed three times every 2 h. Following 24 h of normal culture in an incubator, the hydrogel sponges were digested with a 50 mM EDTA solution to release the cells. The collected cells were then subjected to a live/dead staining assay to assess their viability and recovery success. M2 macrophages cultured under standard conditions served as the control group for comparison.

### 4.4. Application of M2 Macrophage-Hydrogel Sponge in Diabetic Wound Healing

#### 4.4.1. Model Establishment of Chronic Wound

The study was conducted in compliance with the guidelines of the Animal Ethics and Welfare Committee (AEWC) of HZNU (Approval No. HSD-20230828-02) and the National Research Council’s Guide for the Care and Use of Laboratory Animals.

The back hairs of the db/db mice (male, 8 weeks old, body weight of about 30 g) were shaved, completely removed with depilatory cream, and disinfected with alcohol. After one day of recovery, a circular full-thickness excision wound of 8 mm in diameter was made on the skin of the mouse’s back each mouse without penetrating the fascia using a tissue puncher. After wound creation, mice recovered in plastic cages and were administered 4 h later. The blank group did not receive any treatment (Control, *n* = 3), the control group was treated with PBS-treated 2%Alg hydrogel sponge (2%Alg, *n* = 3), and the experimental group was treated with 2%Alg hydrogel sponge embedded with M2 macrophages (2%Alg + M2, *n* = 3). All groups were further bandaged with gauze and 3 M dressing for wounds. The mice were observed and the hydrogel sponge was replaced in day 3, 5, 7, 9,11. The body weight of the mice was recorded, photographs were taken to record the wound healing process. The size of the wound was determined quantitatively using ImageJ software (Version 1.53e). Subsequently, the wound healing rate was calculated based on the following formula:

Wound healing rate (%) = (A_0_ − A_t_)/A_0_

A_0_ and A_t_ in the formula are the initial wound area on day 0 and the wound area on a designated date, respectively.

#### 4.4.2. Histological Analysis

On days 7 and 11 of treatment, mice were anesthetized with 1.25% tribromoethanol. Skin tissue (~1 cm diameter) surrounding the wound was excised and fixed in 4% paraformaldehyde for paraffin embedding. Sections were prepared for HE staining, Masson staining, and Sirius red staining. To assess angiogenesis, tissue sections were immunohistochemically stained for CD31 and α-SMA. Additional unfixed skin tissue samples were used for RNA extraction using the Trizol method. RT-qPCR was performed to analyze the tissue expression of IL-6, TNF-α, TGF-β, IL-10, VEGF, Col1a1, and Col3a1.

### 4.5. Statistical Methods

The fluorescence intensity of confocal microscopy images and wound healing area were analyzed with ImageJ software (Version 1.53e). All experimental data were analyzed statistically using GraphPad Prism 9.0. Measurement data were subjected to either a T-test or one-way ANOVA, as appropriate. The results are presented as the mean ± standard deviation (SD). Statistical significance was determined using a *p*-value threshold of 0.05. Asterisks denote significance levels: * indicates *p* < 0.05; ** indicates *p* < 0.01; *** indicates *p* < 0.001; **** indicates *p* < 0.0001; NS indicates no significant difference.

## Data Availability

All data and materials are available on request from the corresponding author. The data are not publicly available due to ongoing research using a part of the data.

## Supplementary Materials

The following supporting information can be downloaded at: https://www.mdpi.com/article/10.3390/gels11070502/s1, Figure S1: Sterility test of hydrogel sponges; Figure S2: Living/dead cell staining of fibroblasts treated with different hydrogel sponge extracts for 24 h. The scale bars are 100 μm; Figure S3: Living/dead cell staining of fibroblasts treated with different hydrogel sponge extracts for 48 h. The scale bars are 100 μm; Figure S4: M2 polarization of macrophages. (A) Optical photograph of M0 macrophage. (B) Optical photograph of M2 macrophage. (C) The gene expression levels of Arg-1, (*n* = 3, mean ± SD). ** *p* < 0.01; Figure S5: The viability of M2 macrophages in hydrogel sponges. (A) Day1, (*n* = 3, mean ± SD). (B) Day2, (*n* = 3, mean ± SD). (C) Day4, (*n* = 3, mean ± SD). (D) Day6, (*n* = 3, mean ± SD). (*n* = 3, mean ± SD), ** *p* < 0.01, *** *p* < 0.001, **** *p* < 0.0001; Figure S6: The gene expression levels of iNOS, Arg-1, IL-10, TGF-β and VEGF, (*n* = 3, mean ± SD). *** *p* < 0.001, **** *p* < 0.0001, and ns for no significant difference; Figure S7: Sirius Red staining. The scale bars are 500 μm; Figure S8: Area of type I collagen and type III collagen in wound. (A) Collagen area on the seventh day, (*n* = 3, mean ± SD). ** *p* < 0.01, and ns for no significant difference; Figure S9: The gene expression levels of IL-10, IL-6, TNF-α, TGF-β, VEGF, Col3a1 and Col1a1 in skin tissue of mice treated with hydrogel for 7 days, (*n* = 3, mean ± SD). * *p* < 0.05, *** *p* < 0.001, **** *p* < 0.0001, and ns for no significant difference; Figure S10: The gene expression levels of IL-10, IL-6, TNF-α, TGF-β, VEGF, Col3a1 and Col1a1 in skin tissue of mice treated with hydrogel for 11 days, (*n* = 3, mean ± SD). * *p* < 0.05, ** *p* < 0.01, **** *p* < 0.0001, and ns for no significant difference. (B) Collagen area on the eleventh day, (*n* = 3, mean ± SD). * *p* < 0.05, and ns for no significant difference; Table S1: H-score Scale of CD31 Expression in Mice Wound; Table S2: H-score Scale of alpha-SMA Expression in Mice Wound; Table S3: Primers in RT-qPCR; Table S4: Antibodies and ELISA kit in this work.
