# Supplementary material for: Sodium Alginate Hydrogel Sponges Embedded with M2 Macrophages: An Adoptive Cell Therapy Strategy for Accelerated Diabetic Wound Healing
Source: Gels. 2025 Jun 27;11(7):502. doi: 10.3390/gels11070502 (PMC12294235; doi:10.3390/gels11070502)
Supplement: Supplementary file 1 [file gels-11-00502-s001.zip › gels-3681682-supplementary.pdf]

Supporting information

Sodium alginate hydrogel sponges embedded with M2 macrophages: an adoptive therapy strategy for accelerated diabetic wound healing

Qingchang Tian[1,2]*, Wenqi Li[1,2], Lijiaqi Zhang[1,2], Kefen Gan[1,2], Yiting Zhang[1,2], Shuling Wang[1,2,3]*

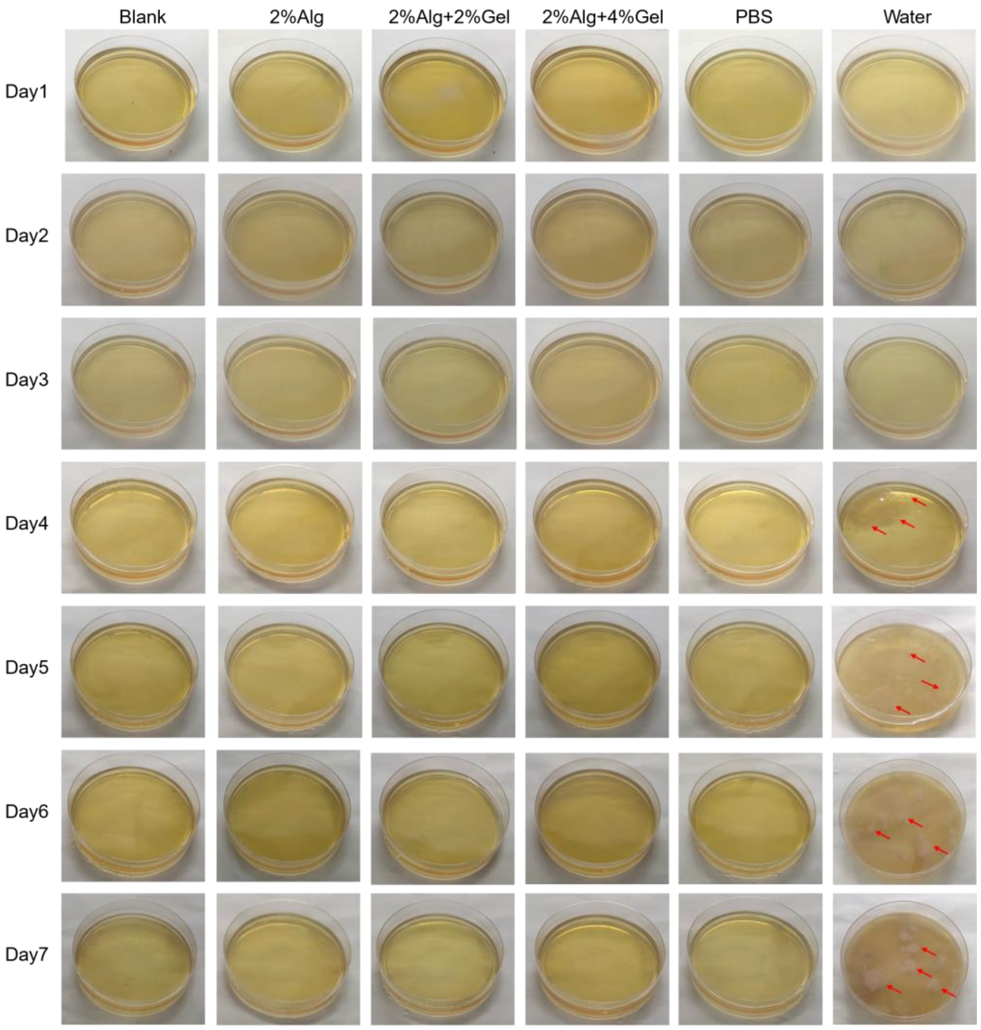

Figure S1 Sterility test of hydrogel sponges

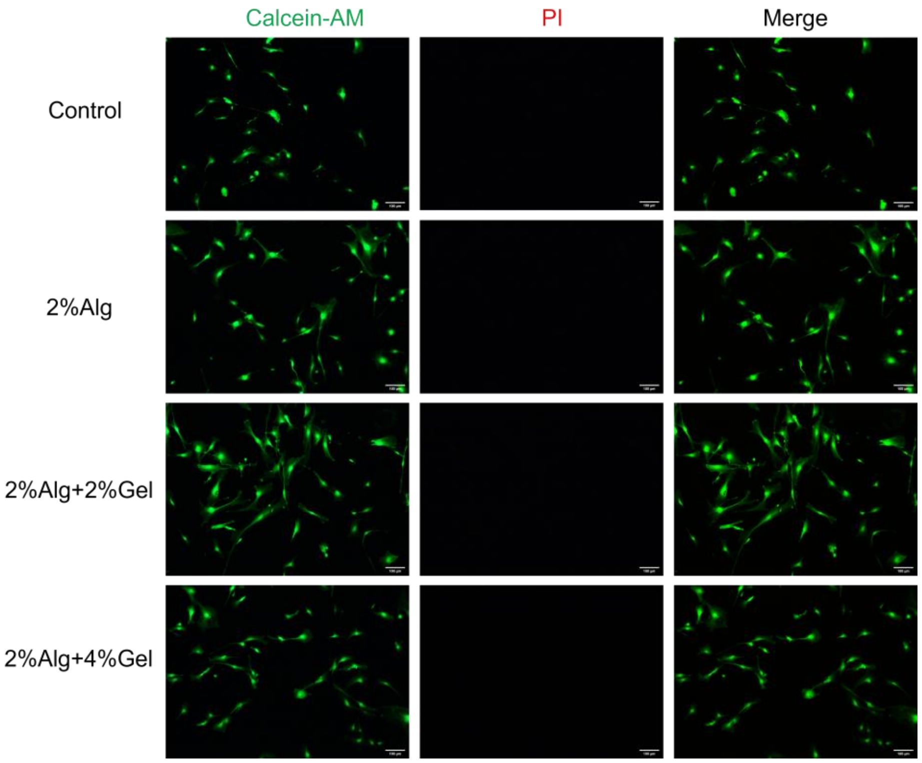

Figure S2 Living/dead cell staining of fibroblasts treated with different hydrogel sponge extracts for 24 h. The scale bars are 100 μm.

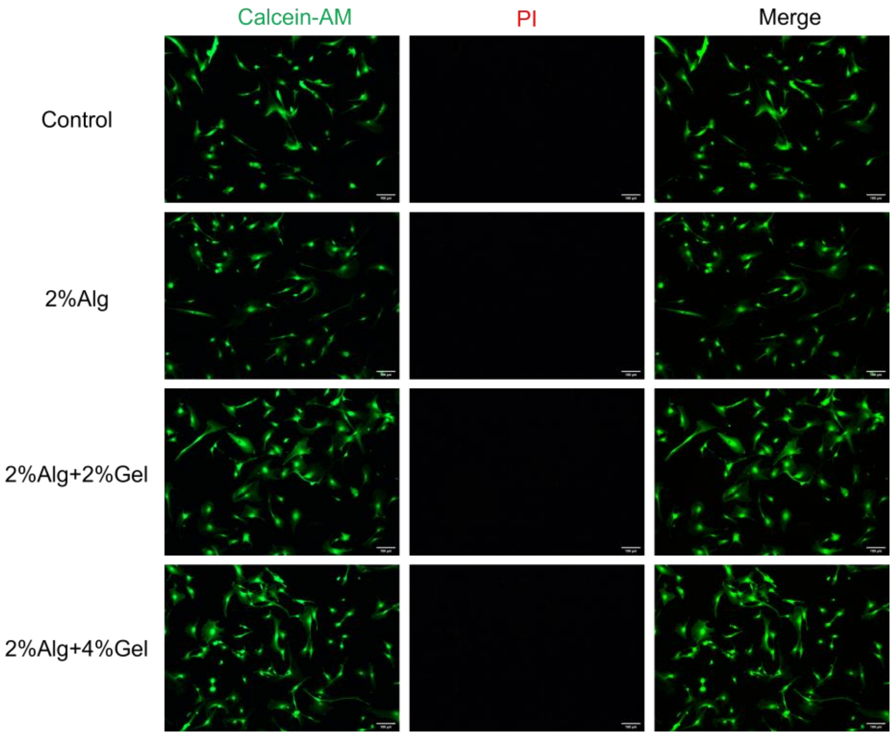

Figure S3 Living/dead cell staining of fibroblasts treated with different hydrogel sponge extracts for 48 h. The scale bars are 100 μm.

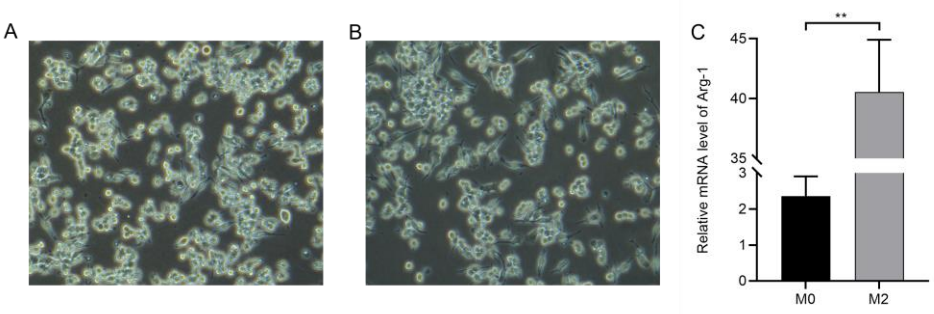

Figure S4 M2 polarization of macrophages. (A) Optical photograph of M0 macrophage. (B) Optical photograph of M2 macrophage. (C) The gene expression levels of *Arg-1*, (n=3, mean ± SD). ***P*<0.01.

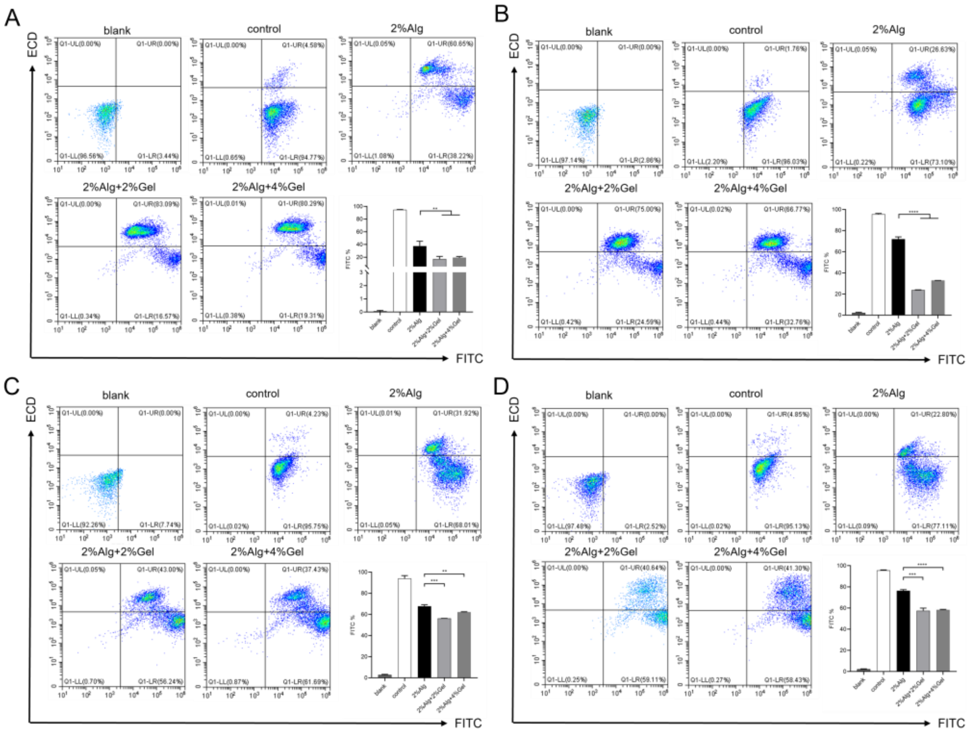

Figure S5 The viability of M2 macrophages in hydrogel sponges. (A) Day1, (n=3, mean ± SD). (B) Day2, (n=3, mean ± SD). (C) Day4, (n=3, mean ± SD). (D) Day6, (n=3, mean ± SD). (n = 3, mean ± SD), **P < 0.01, ***P < 0.001, ****P < 0.0001.

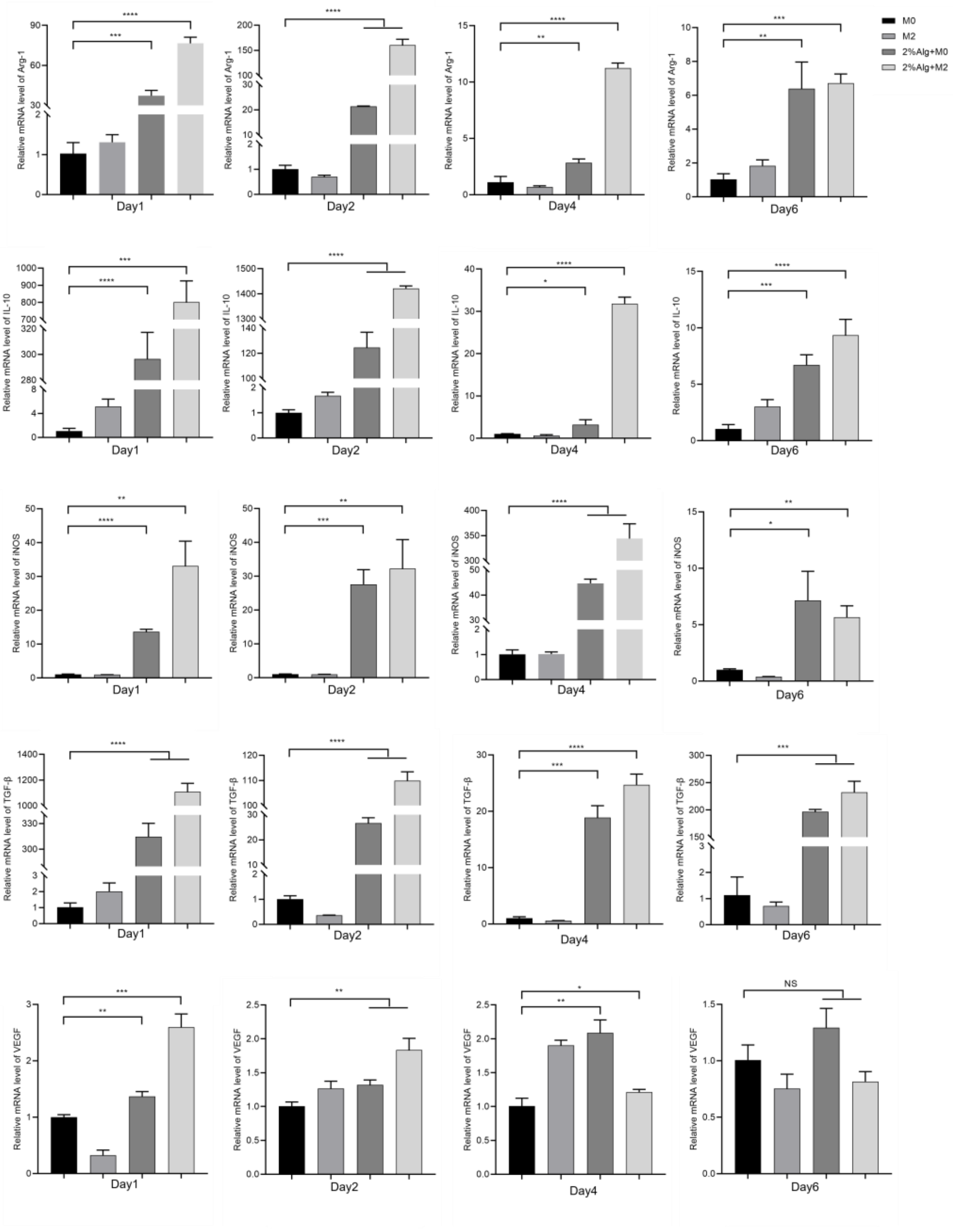

Figure S6 The gene expression levels of *iNOS, Arg-1*, *IL-10, TGF-β* and *VEGF*, (n = 3, mean ± SD). ****P* < 0.001, *****P* < 0.0001, and ns for no significant difference.

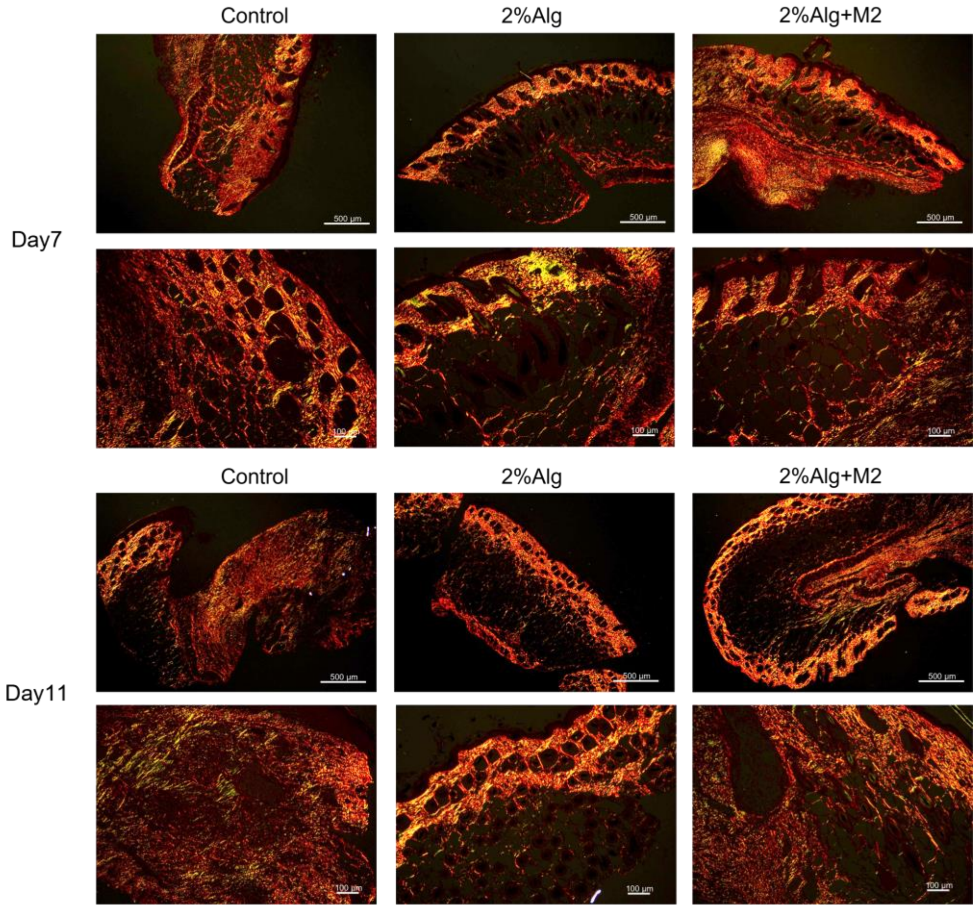

Figure S7 Sirius Red staining. The scale bars are 500 μm.

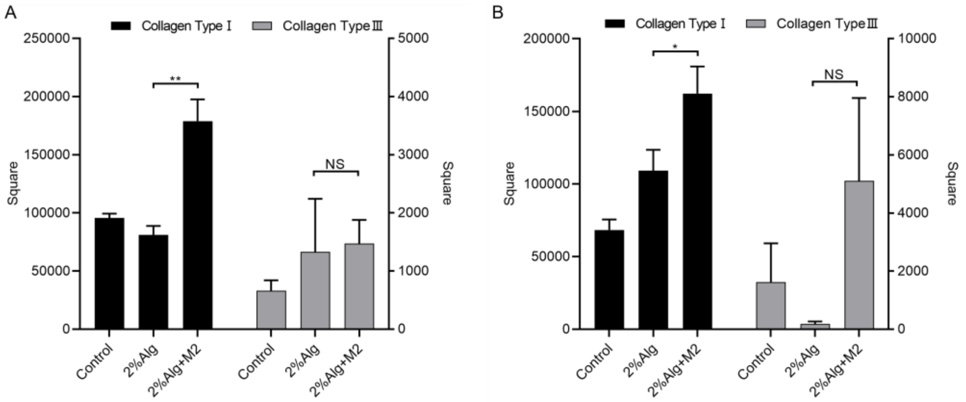

Figure S8 Area of type I collagen and type III collagen in wound. (A) Collagen area on the seventh day, (n=3, mean ± SD). ***P*<0.01, and ns for no significant difference.

(B) Collagen area on the eleventh day, (n=3, mean ± SD). **P*<0.05, and ns for no significant difference.

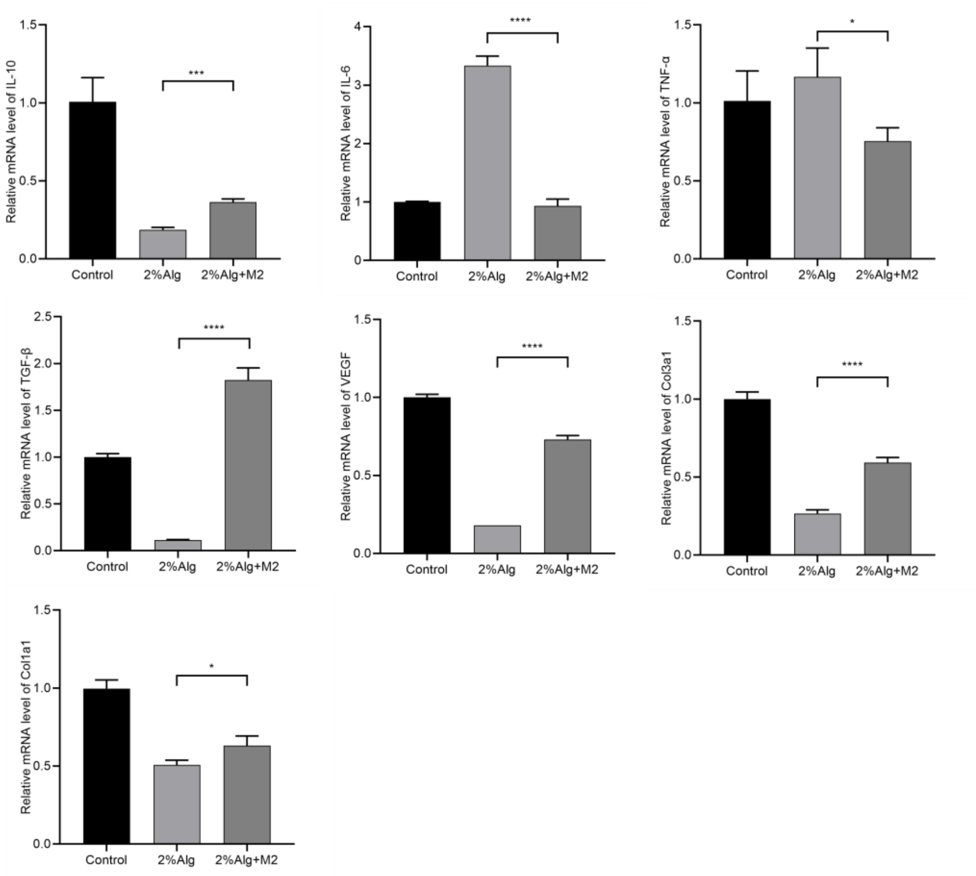

Figure S9 The gene expression levels of *IL-10,* IL-6, TNF-α, *TGF-β*, *VEGF*, *Col3a1* and *Col1a1* in skin tissue of mice treated with hydrogel for 7 days, (n = 3, mean ± SD). **P* < 0.05, ****P* < 0.001, *****P* < 0.0001, and ns for no significant difference.

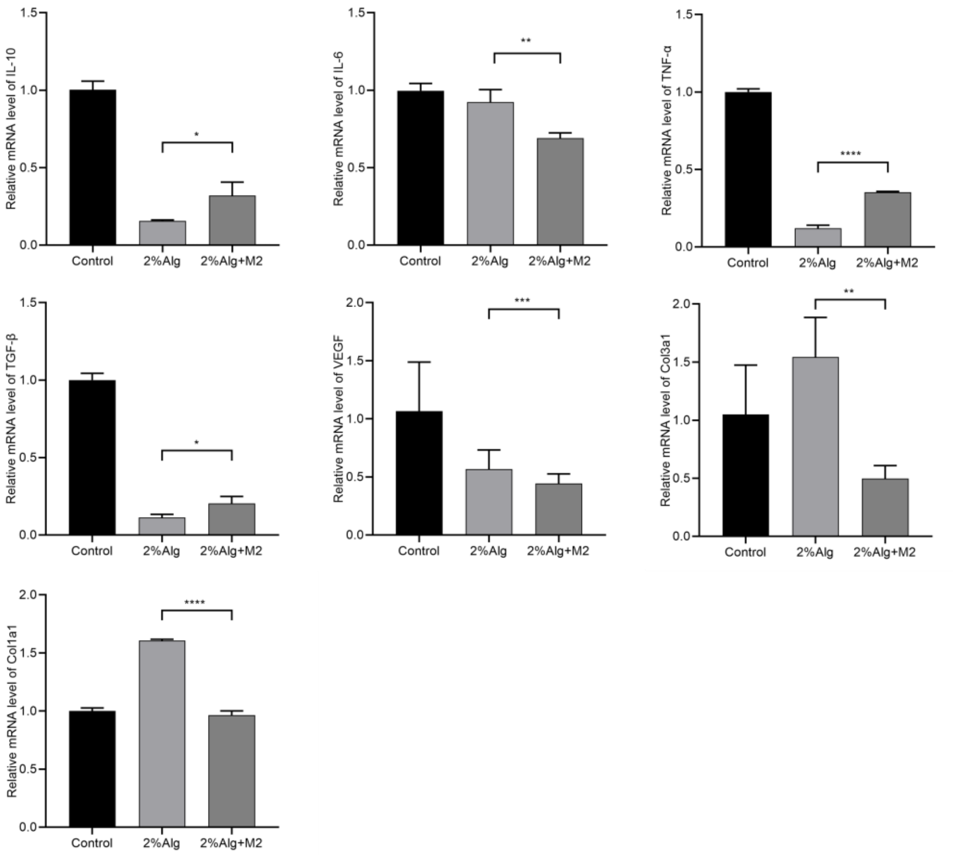

Figure S10 The gene expression levels of *IL-10,* IL-6, TNF-α, *TGF-β*, *VEGF*, *Col3a1* and *Col1a1* in skin tissue of mice treated with hydrogel for 11 days, (n = 3, mean ± SD). **P* < 0.05, ***P* < 0.01, *****P* < 0.0001, and ns for no significant difference.

Table S1 H-score Scale of CD31 Expression in Mice Wound

Time	Name	Neg Nuclei	1+ Nuclei	2+ Nuclei	3+ Nuclei	H-Score	Total Nuclei	Positive Percentage
Day7	Control	31998	1171	943	568	13.73	34680	7.73%
	2%Alg	45828	1799	2741	2621	28.58	52989	13.51%
	2%Alg+M2	29861	5235	5813	5629	72.52	46538	35.84%
Day11	Control	28680	692	828	1417	20.87	31617	9.29%
	2%Alg	25626	1212	1981	1056	27.92	29875	14.22%
	2%Alg+M2	41142	9149	7946	5706	65.93	63943	35.66%

Table S2 H-score Scale of alpha-SMA Expression in Mice Wound

Time	Name	Neg Nuclei	1+ Nuclei	2+ Nuclei	3+ Nuclei	H-Score	Total Nuclei	Positive Percentage
Day7	Control	28370	1814	1736	3782	46.59	35702	20.54%
	2%Alg	35232	3738	5156	7630	71.37	51756	31.93%
	2%Alg+M2	22319	6322	6735	9712	108.52	45088	50.50%
Day11	Control	22226	2064	2557	4918	69.04	31765	30.03%
	2%Alg	19648	1236	1890	3141	55.72	25915	24.18%
	2%Alg+M2	41247	8301	6440	6850	66.41	62838	34.36%

Table S3 Primers in RT-qPCR

Gene	Forward primers(5'-3')	Reverse primers(5'-3')
GAPDH	TGTGTCCGTCGTGGATCTGA	TTGCTGTTGAAGTCGCAGGAG
Arg-1	AGCTCTGGGAATCTGCATGG	ATGTACACGATGTCTTTGGCAGATA
VEGF	ATCGAGTACATCTTCAAGCCAT	GTGAGGTTTGATCCGCATAATC
IL-10	GCCAGAGCCACATGCTCCTA	GATAAGGCTTGGCAACCCAAGTAA
TGF-β	CAAGCTGAACTTGAGCGAGGA	TTTACTCAGTGCCAGAAGCTGGA
iNOS	CAAGCACATTTGGGAATGGAGA	CAGAACTGAGGGTACATGCTGGAG
IL-6	ACAAAGCCAGAGTCCTTCAGAG	GGCAGAGGGGTTGACTT
TNF-α	GACGTGGAACTGGCAGAAGAG	GCCACAAGCAGGAATGAGAG
Col1a1	AAAGATGGACTCAACGGTCTC	CATCGTGAGCCTTCTCTTGAG
Col3a1	TGAAGGGCAGGGAACAACTTGATG	GGATGAAGCAGAGCGAGAAGTAGC

Table S4. Antibodies and ELISA kit in this work.

Name	Source
Rabbit multiclonal [RM1006] to CD31	Abcam, ab281583
Rabbit multiclonal to a-SMA	Proteintech, 80008-1-rr
Mouse VEGF ELISA Kit	Beyotime, PV957
